# Pseudogenization of the chloroplast threonine (*trn*T-GGU) gene in the sunflower family (Asteraceae)

**DOI:** 10.1038/s41598-021-00510-4

**Published:** 2021-10-26

**Authors:** Abdullah, Furrukh Mehmood, Parviz Heidari, Abdur Rahim, Ibrar Ahmed, Peter Poczai

**Affiliations:** 1 Department of Biochemistry, Faculty of Biological Sciences, Quaid-i-Azam University, Islamabad, 45320 Pakistan; 2 Faculty of Agriculture, Shahrood University of Technology, 3619995161 Shahrood, Iran; 3 Government Degree College Nowshera, Abdul Wali Khan University, Mardan, KPK Pakistan; 4 Alpha Genomics Private Limited, Islamabad, 45710 Pakistan; 5 Finnish Museum of Natural History, University of Helsinki, P.O. Box 7, 00014 Helsinki, Finland; 6 Faculty of Biological and Environmental Sciences, University of Helsinki, P.O. Box 65, 00065 Helsinki, Finland

**Keywords:** Evolutionary genetics, Molecular evolution, Phylogenetics

## Abstract

The chloroplast genome evolves through the course of evolution. Various types of mutational events are found within the chloroplast genome, including insertions-deletions (InDels), substitutions, inversions, gene rearrangement, and pseudogenization of genes. The pseudogenization of the chloroplast threonine (*trn*T*-*GGU) gene was previously reported in *Cryptomeria japonica* (Cupressaceae), *Pelargonium* × *hortorum* (Geraniaceae), and *Anaphalis sinica* and *Leontopodium leiolepis* of the tribe Gnaphalieae (Asteroideae, Asteraceae). Here, we performed a broad analysis of the *trn*T-GGU gene among the species of 13 subfamilies of Asteraceae and found this gene as a pseudogene in core Asteraceae (Gymnarrhenoideae, Cichorioideae, Corymbioideae, and Asteroideae), which was linked to an insertion event within the 5′ acceptor stem and is not associated with ecological factors such as habit, habitat, and geographical distribution of the species. The pseudogenization of *trn*T-GGU was not predicted in codon usage, indicating that the superwobbling phenomenon occurs in core Asteraceae in which a single transfer RNA (*trn*T-UGU) decodes all four codons of threonine. To the best of our knowledge, this is the first evidence of a complete clade of a plant species using the superwobbling phenomenon for translation.

## Introduction

The plant family Asteraceae (Compositae), commonly known as the daisy or sunflower family, is among the three megadiverse families that comprise up to 25% of angiosperm species^1^. The Asteraceae family has between 25,000 and 35,000 species which is ~ 10% of flowering plants and comparable only to the Fabaceae and Orchidaceae families^1^. These species are diverse in distributions and habitat, exist on every continent, including Antarctica, and occupy every type of habitat^1,2^. This family is divided into 13 subfamilies, including Barnadesioideae, Famatinanthoideae, Stifftioideae, Mutisioideae, Gochnatioideae, Wunderlichioideae, Hecastocleidoideae, Pertyoideae, Carduoideae, Gymnarrhenoideae, Cichorioideae, Corymbioideae, and Asteroideae^1,3,4^. Among these families, the four subfamilies Gymnarrhenoideae, Cichorioideae, Corymbioideae, and Asteroideae are considered core Asteraceae^5^. The subfamily Asteroideae is the youngest and largest subfamily of Asteraceae, comprising more than 17,000 species^1,4^.

The chloroplast is a vital organelle in plants due to its role in photosynthesis^6^. It is prokaryotic in origin and shows uniparental inheritance—paternal in some gymnosperms and maternal in most angiosperms^7–9^. The uniparental inheritance and variable mutation rate of different regions of the chloroplast genome make it suitable for studies ranging from population genetics to phylogenetics^10,11^. Many mutational events occur in chloroplast genomes, including InDels (Insertions-deletions), substitutions, inversions, and copy-number variations^12–15^. Some of these mutational events also lead to complete deletion or pseudogenization of genes within the chloroplast genome, including protein-coding genes and transfer RNA genes^16–18^. Pseudogenization is a process by which a functional gene becomes non-functional. Pseudogenes have significant homology to functional genes but disruptive mutations led to lost of  function often via formation of truncated proteins^19,20^. Pseudogenes reflect the evolutionary past^19^, thus being important elements shaping genome content^21^. Pseudogenization has been linked to gene size and shown to occur more frequently in larger genes encoding a protein product of up to 1000 amino acids^22^. It can also be linked to ecological niches such as utilization of resources related to energy, metabolism, interaction among organisms, host-specific responses, and lifestyle of the organism^19,21^.

Two transfer RNA genes exist for threonine, located in the large single-copy region of the chloroplast. One copy of threonine (*trn*T-GGU) lies between protein-coding genes *atp*A and *psb*D along with *trn*G-UCC and *trn*R-UCU. Another copy of threonine (*trn*T-UGU) is located between *rps*4 and *trn*L-UAA near the *trn*T-F region, which is widely used in phylogenetic analyses and barcoding studies^4,23^. Based on the wobbling rule^24^, threonine is encoded by four codons during translation in which the two codons ACC and ACU are decoded by *trn*T-GGU, whereas the two remaining codons ACA and ACG are decoded by *trn*T-UGU. However, a study based on functional analysis of the plastid genome of tabacum, after developing mutant lines, indicates that the *trn*T-UGU is able to degenerate all four codons of threonine, making *trn*T-GGU unessential^25^ for translation of threonine. The pseudogenization of *trn*F-GAA has also been reported in some plant lineages^17,26,27^. Previously, the pseudogenization of *trn*T-GGU has been reported in *Cryptomeria japonica* D. Don. (family Cupressaceae)^28^, *Pelargonium* × *hortorum* (family Geraniaceae)^29^, and *Anaphalis sinica* and *Leontopodium leiolepis* of the tribe Gnaphalieae (Asteroideae (Asteraceae)^30^. Here, we are interested in determining the range of *trn*T-GGU pseudogenization in the family Asteraceae, its possible mechanism of pseudogenization, and the process of codon degeneration in its absence. To the best of our knowledge, we for the first time analyzed the *trn*T-GGT genes in 134 representative species of Asteraceae belonging to 13 subfamilies, which were diverse in habit and habitat and included 97 species of Asteroideae (Table S1). We report that *trn*T-GGT is either absent or a pseudogene in core Asteraceae due to an insertion event in the 5′ acceptor stem. Moreover, codon usage analysis indicates that superwobbling may be a possible mechanism by which species decode all four codons using *trn*T-UGU in the absence/pseudogenization of *trn*T-GGU.

## Results

### Analysis of *trn*T-GGU among species of Asteraceae

**Figure 1:**
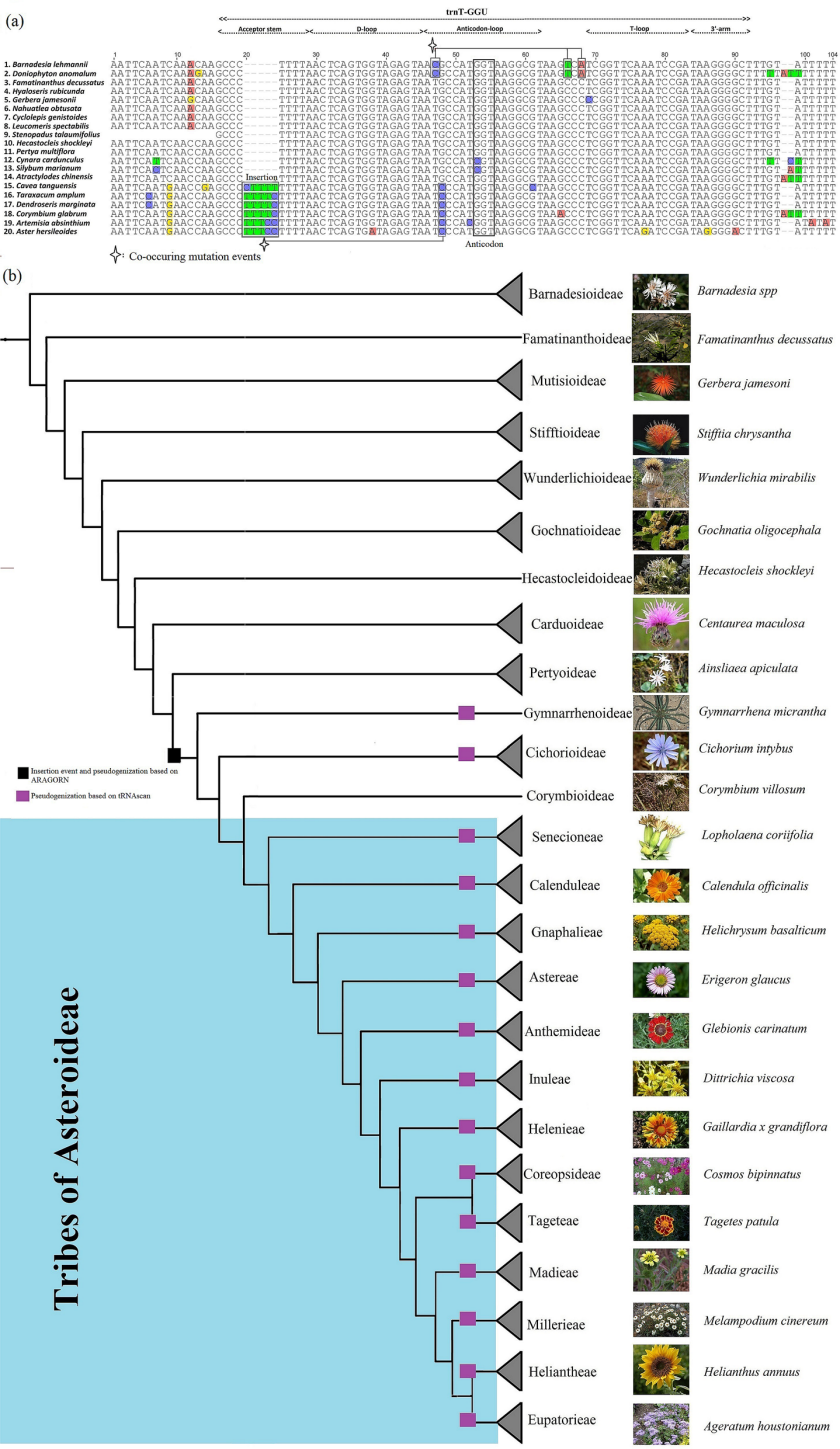
Multiple sequence alignment of the plastid threonine (*trn*T-GGU) gene and the position of pseudogenes within the phylogenetic tree. (**a**) All functional parts of the gene have been noted above the alignment. The insertion occurring in the acceptor stem is highlighted. Co-occurrence of mutational events in some species are shown above and below the alignment. **(b)** The black block indicates the starting node of the insertion event in the 5′ acceptor stem and pseudogene detected by ARAGORN among species of the ‘Core Asteraceae’ clade, whereas the purple block indicates the presence of pseudogenes based on tRNAscan-SE. Leaves of the phylogenetic tree from Barnadesioideae to Corymbioideae represent subfamilies of Asteraceae, while all other leaves of phylogenetic trees represent 13 tribes of the Asteroideae subfamily (indicated in blue background), followed by the icon size photo of a representative species used in our analysis. The species from 13 tribes of Asteroideae were included in the analysis and their names are noted at each node in the highlighted background. The representative photos of each subfamily and tribe are included in the figure and the species names are provided in front of each photo.

**Figure 2:**
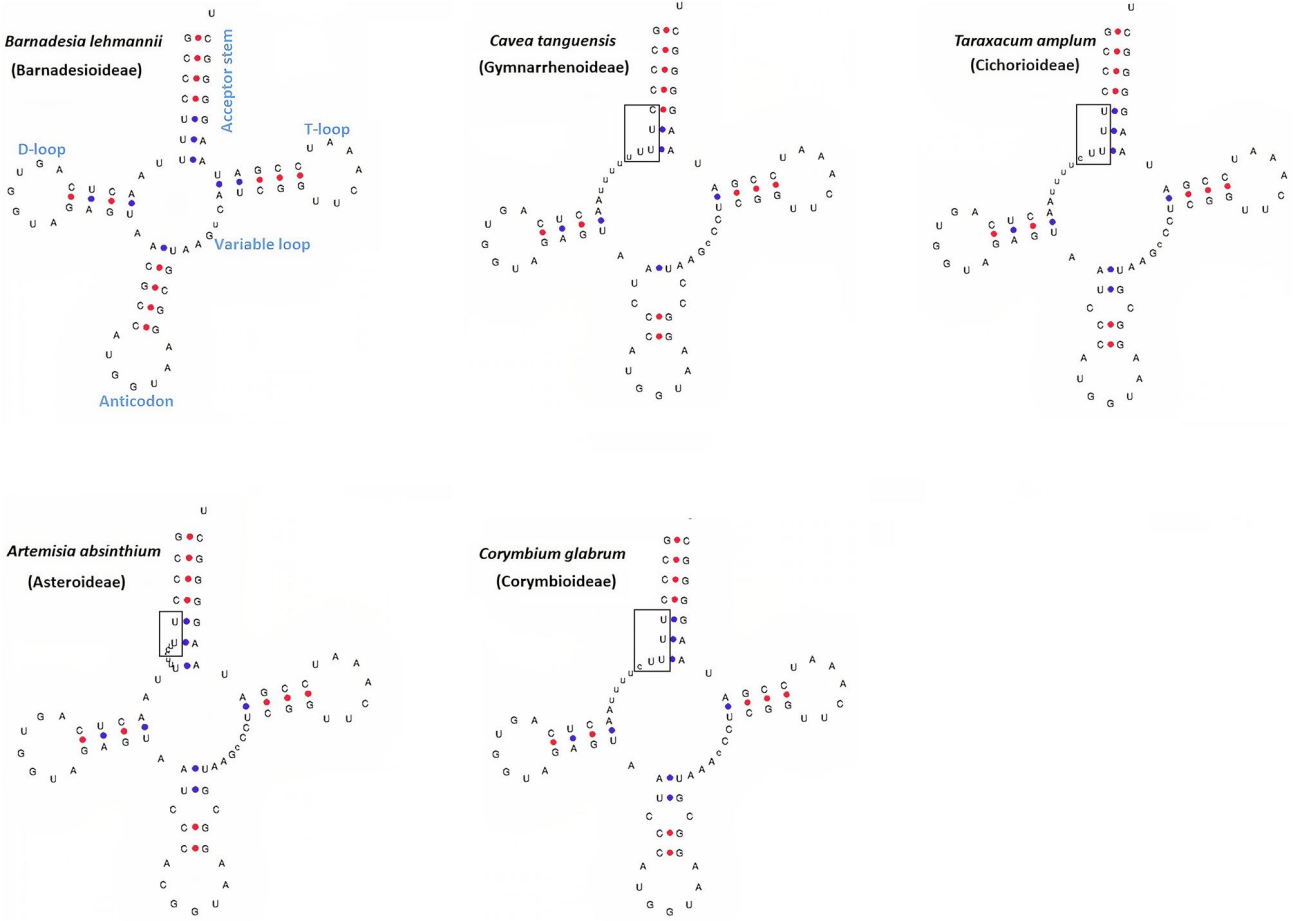
Structure of *trn*T-GGU gene of species of 13 subfamilies. One species was taken from each subfamily of core Asteraceae. The *trn*T-GGU gene of *Barnadesia lehmannii* is labeled to show the functional parts as representative of all species. The perfect clover leaf structure of *trn*T-GGU exists in the species of nine subfamilies, including Barnadesioideae, Famatinanthoideae, Stifftioideae, Mutisioideae, Gochnatioideae, Wunderlichioideae, Hecastocleidoideae, Pertyoideae, and Carduoideae. The *B. lehmannii* represent the structure of *trn*T-GGU of the species of all nine subfamilies. The insertion occurred in the species of four subfamilies of core Asteraceae (Gymnarrhenoideae, Cichorioideae, Corymbioideae, and Asteroideae), which also correspond to mismatches above the anticodon loop. The insertion is highlighted with a box.

We compare the *trn*T-GGU gene among 13 subfamilies of Asteraceae. The analyses revealed an insertion event (i.e., CTTTT/TTTTC/TTTCC) at the 5′ acceptor stem of the *trn*T-GGU gene in core Asteraceae, while this was lacking in the species of other subfamilies of Asteraceae (Fig. 1a,b). This insertion event was found to be linked to the pseudogenization of the *trn*T-GGU in all subfamilies of core Asteraceae based on the result of ARAGORN, as the gene was not annotated in any species. However, the gene was found to be non-functional in three subfamilies of core Asteraceae, as a functional copy was predicted in single species of Corymbioideae with low infernal score based on the result of tRNAscan-SE (Fig. 1b). The high infernal score indicates high matching with other tRNAs of the database and reflects high accuracy of the predicted tRNAs. Therefore, the infernal  program was integrated into tRNAscan-SE to improve performance and prediction accuracy and to achieve a better functional classification of tRNA. The infernal score of the *trn*T-GGU gene ranged from 49.4 to 65 in the species of those subfamilies that lacked the insertion event (Table 1), indicating that the gene is completely functional in these species. In contrast, tRNAscan-SE detected mismatch isotypes of the *trn*T-GGU gene in Gymnarrhenoideae, with a low infernal score and pseudogene in Cichorioideae, functional copy with low infernal score in Corymbioideae, and diverse types of results in the subfamily of Asteroideae (Table S2). The structure of the *trn*T-GGU gene of the species of each subfamily showed that the mismatch/mismatches is/are present in the species of Gymnarrhenoideae, Cichorioideae, Corymbioideae, and Asteroideae mostly at the 5′ acceptor stem and anticodon loop, whereas the species that lacked the aforementioned insertion have a complete cloverleaf structure (Figs. 2, S1). These data suggest that the pseudogenization event might be widespread in the Asteraceae family and may be limited to core Asteraceae.

**Table 1:** Prediction of *trn*T-GGU genes in the representative species of 13 subfamilies.

Serial number	Species	tRNAscan prediction	Infernal score	Isotype	Anticodon	Subfamily
1	*Doniophyton anomalum*	Thr	65.8	Thr	GGU	Barnadesioideae
2	*Barnadesia lehmannii*	Thr	65.8	Thr	GGU	Barnadesioideae
3	*Famatinanthus decussatus*	Thr	57.1	Thr	GGU	Famatinanthoideae^£^
4	*Hyaloseris rubicunda*	Thr	57.1	Thr	GGU	Stifftioideae^£^
5	*Gerbera jamesonii*	Thr	49.4	Thr	GGU	Mutisioideae
6	*Cyclolepis genistoides*	Thr	57.1	Thr	GGU	Gochnatioideae^£^
7	*Nahuatlea obtusata*	Thr	57.1	Thr	GGU	Gochnatioideae^£^
8	*Leucomeris spectabilis*	Thr	57.1	Thr	GGU	Wunderlichioideae^£^
9	*Stenopadus talaumifolius*	Thr	57	Thr	GGU	Wunderlichioideae^£^
10	*Hecastocleis shockleyi*	Thr	57.1	Thr	GGU	Hecastocleidoideae^£^
11	*Pertya multiflora*	Thr	57.1	Thr	GGU	Pertyoideae
12	*Atractylodes chinensis*	Thr	57.1	Thr	GGU	Carduoideae
13	*Cynara cardunculus*	Thr	57	Thr	CGU	Carduoideae
14	*Silybum marianum*	Thr	57	Thr	CGU	Carduoideae
15	*Cavea tanguensis*	tRNA*	32.7	Ile2	GGU	Gymnarrhenoideae^£^
16	*Dendroseris berteroana*	Thr	34.6	Thr	GGU	Cichorioideae
17	*Taraxacum amplum*	Thr	34.6	Thr	GGU	Cichorioideae
18	*Corymbium glabrum*	Thr	32.6	Thr	GGU	Corymbioideae^£^
19	*Artemisia ordosica*	tRNA*	27.8	Ile2	GGU	Asteroideae
20	*Aster hersileoides*	Not detected	N/A	N/A	GGU	Asteroideae

^£^transfer RNA sequence extracted from raw read of Sequence Read Archive (SRA) of NCBI.

* tRNAs with mismatch isotypes.

### Analyses of *trn*T-GGU genes among the species of Carduoideae

The analyses of 11 species from 11 different genera of Carduoideae showed that the functional *trn*T-GGU gene with a high infernal score ranged from 55.7 to 57.1 (Table S3). Except for *Atractylodes chinensis* (DC.) Koidz, the analyses of the other ten species revealed the presence of anticodon CGU (Fig. S2). We also found an insertion (CTCAG) in the D-loop of *Saussurea inversa* Raab-Straube, which slightly decreases the infernal score to 55.7. The structure analyses support the presence of all functional parts of the gene in the species of Carduoideae (Fig. S1).

### Analyses of *trn*T-GGU genes among the species of Cichorioideae

**Figure 3:**
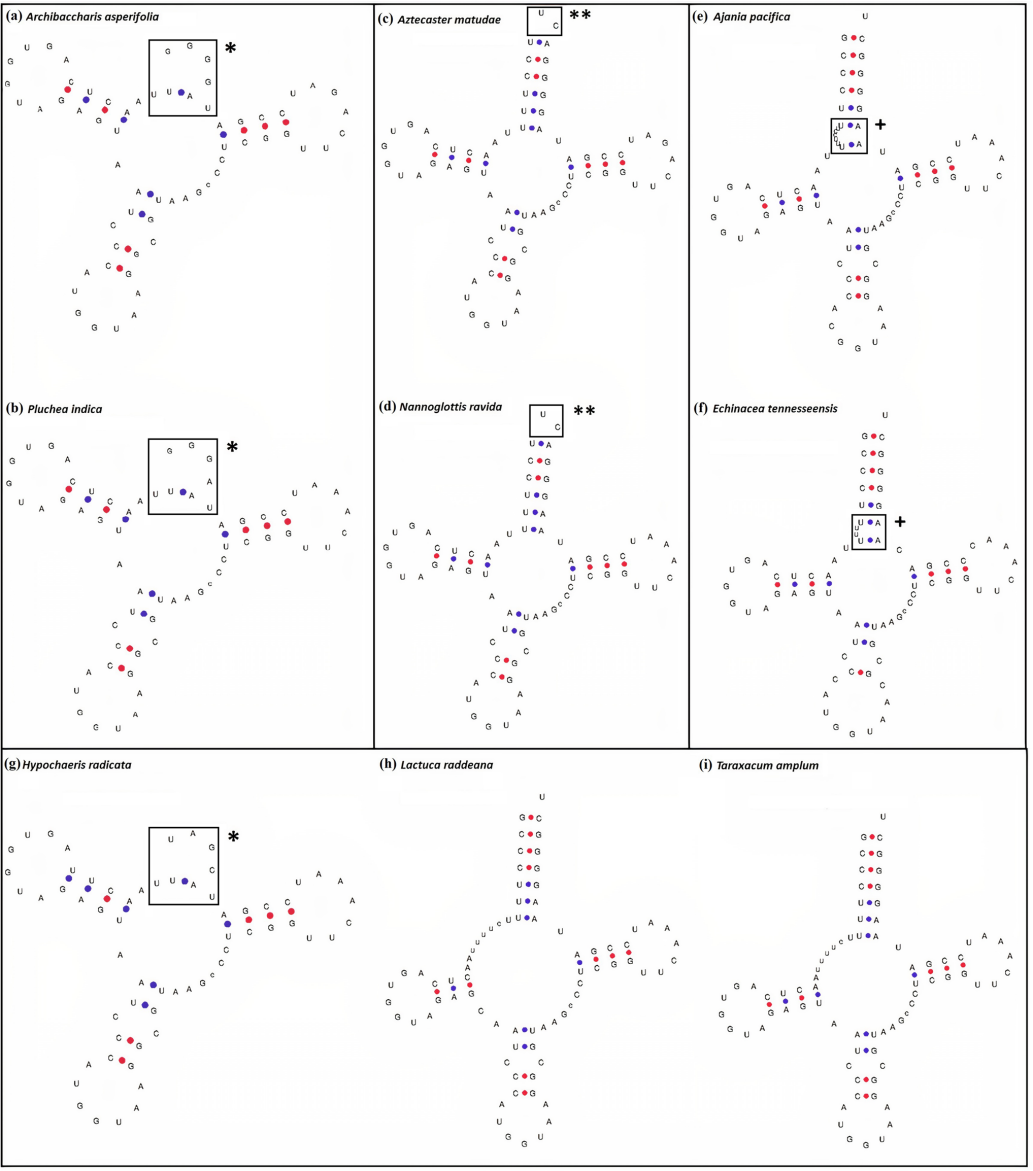
Structure of pseudo or low infernal score *trn*T-GGU gene in Asteroideae and Cichorioideae. The structure of the gene from ‘a’ to ‘f’ shows the species of Asteroideae, whereas from ‘g’ to ‘i’ represents species of Cichorioideae. (**a** and **b**) Pseudogenization of the gene occurred due to loss of the acceptor stem. (**c** and **d**) The genes are predicted with low infernal score (22.6) only by tRNAscan-SE and were not predicted by ARAGORN. However, the mismatch at 5′ and 3′ indicates that this gene may also be non-functional. (**e** and **f**) The gene of *trn*T-GGU predicted as mismatch isotypes for isoleucine. The clear insertion is visible in the acceptor stem, which disturbs the cloverleaf structure. (**g**) Pseudogenization of the gene occurred due to loss of the acceptor stem. (**h**) Indicates the tRNAscan predicted pseudo gene. (**i**) Indicates the tRNAscan predicted gene with low infernal score of 34.6. All the species show the mismatch of c–c, which forms an extra loop-like structure above the codon loop. * indicates loss of the acceptor arm, ** indicates mismatch at 5′ and 3′, + indicates the missing of base pair of uridines in the acceptor arm due to insertion.

The analyses of 13 species from 13 genera of Cichorioideae revealed the pseudogenization of the *trn*T-GGU gene in all species based on the result of ARAGORN, whereas the gene was found to be pseudo in four species based on the prediction of tRNAscan-SE. The tRNAscan-SE predicted *trn*T-GGU with mismatch isotypes of lysine along with truncated start and truncated end in *Hypochaeris radicata* L., pseudogene in *Lactuca raddeana* Maxim. (Fig. S3), and the gene was not predicted due to deletion events in *Stebbinsia umbrella* (Franch.) Lipsch. and *Ixeris polycephala* Cass. The structure of the species *H. radicata* and *L. raddeana* showed certain mismatches at the acceptor stem and specific variations in the variable loop (Fig. 3). In other species, the *trn*T-GGU gene was predicted with a low infernal score of 31 to 34.6 (Table S4).

### Analyses of *trn*T-GGU genes among the species of Asteroideae

We analyzed 97 species belonging to 78 genera and 13 tribes of the Asteroideae subfamily. The analyses revealed that the *trn*T-GGU gene exists as a pseudogene in the species of all tribes (Table S4). This gene was not detected by ARAGORN in any species, whereas tRNAscan-SE did not predict this gene in 60 species. The *trn*T-GGU gene was predicted as a pseudogene in 9 species and as mismatch isotypes of isoleucine and lysine in 16 species (Table S2. We also detected this gene with a low infernal score of 22.6 in 12 species. However, the manual analyses of the structure revealed truncation at the 5′ and 3′ ends, indicating that the gene might also be non-functional in these species. The structure of representative species is shown in Fig. 3. The pseudogenization of the *trn*T-GGU gene occurs throughout the Asteroideae subfamily due to a high mutational rate (substitutions and insertion-deletion) in all functional parts of the genes. However, the highest mutations and degradation were recorded in the 5′ acceptor arm and the 3′ arm (Fig. S4). Pseudogenization occurred in the species of Asteroideae irrespective of the habit, habitat, and native range (Table S1, Table S2). We analyzed the *trn*T-GGU gene of 22 species of *Artemisia* L., 21 species of *Aldama* La Llave, and 25 species of *Diplostephium* Kunth to determine the extent of similarities and differences existing within this gene among closely related species (species of same genus). The analyses showed high similarities in the pseudogene of the *trn*T-GGU gene and fewer variations among species of the same genus (Figs. S5, S6, S7).

### Codon usage analysis

The codon usage analysis of five representative species, four (*Artemisia ordosica*, *Aster hersileoides*, *Symphyotrichum subulatum*, and *Helianthus annuus*) of which represent species that lack the *trn*T-GGU gene or contained a pseudo copy, while *Barnadesia lehmannii* had a functional copy of *trn*T-GGU with a high infernal score of up to 65. The aforementioned species revealed high similarities in codon usage for amino acid threonine (Table S5). These findings showed that the pseudogenization of *trn*T-GGU did not cause any alteration in codons of protein-coding sequences and translates proteins similar to the species that have both functional tRNA.

## Discussion

The loss/pseudogenization of the *trn*T-GGU gene was investigated in 13 subfamilies of Asteraceae. Our findings show the loss/pseudogenization of the *trn*T-GGU gene in the species of the four subfamilies of Gymnarrhenoideae, Cichorioideae, Corymbioideae, and Asteroideae (collectively known core Asteraceae) based on the result of ARAGORN, whereas pseudogenization of the *trn*T-GGU gene was predicted in Gymnarrhenoideae, Cichorioideae, and Asteroideae based on the result of tRNAscan-SE. In addition, pseudogenization of the *trn*T-GGU gene was reported in previous studies, including the *Cryptomeria japonica* D. Don. of the family Cupressaceae^28^, *Pelargonium* × *hortorum* of the family Geraniaceae^29^, and *Anaphalis sinica* and *Leontopodium leiolepis* of the tribe Gnaphalieae of Asteroideae (Asteraceae)^30^. The species of the aforementioned subfamilies are diverse in terms of habit, habitat, and geographical distribution (Table S1). This demonstrates that pseudogenization is not linked to convergent evolution or environmental factors and a clade-specific event was found following clear phylogenetic patterns that agreed with the previously established phylogeny of the family Asteraceae^1,4,30^. The pseudogenization was linked to an insertion event in the 5′ acceptor stem. Earlier studies have demonstrated that insertions and deletions generate substitutions^18,31–33^ due to the recruitment of error-prone DNA polymerase^34,35^. Hence, this insertion may increase the rate of mutations of the *trn*T-GGU gene either causing pseudogenization of the gene by affecting the functional parts of the gene or leading to complete deletion of the gene. Previously, loss of the *trn*T-GGU gene was noted to be linked to an inversion event in *Pelargonium* × *hortorum* (Geraniaceae)^29^. A similar large inversion event (22.8 kb) has also been reported in Asteraceae, except for species of Barnadesioideae (earlier diverged clade)^36^. One endpoint of this inversion is located between *trn*S-GCU and *trn*G-UCC genes, whereas the other endpoint is present between *trn*E-UUC and *trn*T-GGU^36^. However, the absence of pseudogenization of *trn*T-GGU in species of subfamilies Famatinanthoideae, Stifftioideae, Mutisioideae, Gochnatioideae, Wunderlichioideae, Hecastocleidoideae, Pertyoideae, and Carduoideae reveals that the inversion event is not responsible for the pseudogenization of the *trn*T-GGU gene. Therefore, the insertion event may be responsible for pseudogenization and provide a plausible explanation for the pseudogenization of *trn*T-GGU.

Insertion-deletion and pseudogenization events are also considered important to gain insight into the evolutionary past^19^ and phylogenetic patterns^37^. Previously, the 9 bp deletion in *ndh*F gene was shared by three subfamilies of core Asteraceae, including Cichorioideae, Corymbioideae, and Asteroideae^38,39^, the 9 bp and 18 bp deletion in *rpo*B gene was shared by the six subfamilies Carduoideae, Pertyoideae, Gymnarrhenoideae, Cichorioideae, Corymbioideae, and Asteroideae^40^, and the 15 bp deletion in *rpo*B was shared by the seven subfamilies Hecastocleidoideae, Carduoideae, Pertyoideae, Gymnarrhenoideae, Cichorioideae, Corymbioideae, and Asteroideae^40^. All of these deletion events in protein-coding genes were used to gain insights into the phylogenetics of Asteraceae. Our result provides new support for the phylogenetic history and evolution of core Asteraceae based on specific insertion events and pseudogenization of the *trn*T-GGU gene, which is limited to core Asteraceae.

The loss/pseudogenization of the *trn*T-GGU gene was not reflected in codon usage analysis, which is in agreement with the previous report on *Pelargonium* × *hortorum* from the family Geraniaceae^29^. However, the conventional wobble rules described by Crick (1966)^24^ suggest the presence of 32 tRNA in the plastid genome and consider both threonine genes essential, *trn*T-GGU for decoding codons ACC and ACU of mRNA, and *trn*T-UGU for decoding codons ACA and ACG of mRNA. Therefore, the wobble rules cannot describe the adapted mechanism of the species of core Asteraceae by which they cover the deficiency of the *trn*T-GGU gene. The functional study of tRNAs indicates that 25 tRNA will be sufficient to decode all 61 codons by using superwobbling phenomena^25^ in which a single tRNA species containing an unmodified uridine in the wobble position of the anticodon can read an entire fourfold degenerate codon box^25,41^. The study of Alkatib et al.^25^ experimentally proved, based on knock-out mutants of tabacum chloroplast, that the *trn*T-UGU followed the superwobbling rule and degenerated all four codons of threonine, thus making *trn*T-GGU nonessential for translation of threonine codons. The deletion/pseudogenization of the *trn*T-GGU gene in the core Asteraceae, specifically subfamily Asteroideae (comprising about 17,000 species^1^) shows pseudogenization of this gene within the representative species of 13 tribes (from early diverged tribe Senecioneae to recently diverged tribe Eupatorieae), suggests that superwobbling may be responsible for the translation of threonine codons, as the species of core Asteraceae did not show any adverse events, and supports the findings of Alkatib et al.^25^ in naturally growing species.

In conclusion, the pseudogenization of the *trn*T-GGU gene occurred in core Asteraceae and is linked to the insertion event in the 5′ acceptor arm. The insertion event provides new insight into the evolution of core Asteraceae and broadens our knowledge of the evolution of the chloroplast genome in angiosperms. The codon usage analysis of the species indicates that superwobbling may be the universal phenomena in core Asteraceae by which they proceed to translate all four codons using only *trn*T-UGU in the absence of *trn*T-GGU.

## Materials and methods

The complete chloroplast genome sequences of 124 species belonging to six subfamilies of Asteraceae were retrieved from the National Center for Biotechnology and Information (NCBI) (Table S1). These included the chloroplast genome sequences of 96 species of Asteroideae, 13 species of Cichorioideae, 11 species of Carduoideae, 2 species of Barnadesioideae, and 1 species each of Mutisioideae and Pertyoideae. The raw reads of 10 other species (Table S1) were retrieved from the Sequence Read Archive (SRA) to extract *trn*T-GGU gene. This enabled us to include the data of seven other subfamilies, including one species each of Gymnarrhenoideae, Corymbioideae, Famatinanthoideae, Hecastocleidoideae, and Stifftioideae and two species each of Gochnatioideae and Wunderlichioideae (Table S1). Moreover, the *trn*T-GGU gene of *Chrysanthemoides incana* (Asteroideae) was also extracted from raw reads to cover the tribe Calenduleae. The raw read of these species was retrieved and mapped to *Silybum marianum* (L.) Gaertn. (KT267161) in Geneious R8.1^42^ using Medium–Low Sensitivity/Fast option, keeping all other parameters as default. The consensus was annotated and extracted after confirmation of mapping quality, specifically focusing on the *trn*T-GGU region. This approach enabled us to include diverse species in our study regarding geographical distribution, habit, and habitat (Table S1). We also retrieved chloroplast genome sequences of 25 species of *Diplostephium*, 22 species of *Artemisia*, and 21 species of *Aldama* to perform comparative analyses of the *trn*T-GGU gene at genus level in the Asteroideae subfamily (Table S6). The pseudogenization of the *trn*T-GGU gene was confirmed by reannotation of the *trn*T-GGU region by ARAGORN v.1.2.38^43^ and tRNAscan-SE v.2.0.7^44^ whereas the infernal score was calculated for each tRNA using Infernal v.1.1^45^ integrated in tRNAscan. The prediction of ARAGORN and/or tRNAscan-SE v.2.0.7 was recorded for each species.

The structural variations within *trn*T-GGU were analyzed by utilizing multiple alignment tool using clustalW^46^ integrated into Geneious R8.1 and inspected manually at the family, subfamily, and genus levels. To determine the position of pseudogenes on the phylogenetic tree, we drew a representative phylogenetic tree based on the previously reported data set of Panero et al.^4^ by running IQ-Tree with settings reported in Mehmood et al.^11^ while a high-quality representative tree was drawn using Integrative Tree Of Life (iTOL v.4.0)^47^.

We analyzed codon usage of protein-coding genes in five representative species to examine the effect of pseudogenization of *trn*T-GGU on the sequences of protein-coding genes. We included four species: *Artemisia ordosica* (contained tRNAs with mismatch isotypes of isoleucine with infernal score 27.8 instead of *trn*T-GGU gene), *Aster hersileoides* (copy of trnT-GGU gene not predicted by ARAGORN and tRNAscan-SE), *Symphyotrichum subulatum* (*trn*T-GGU gene predicted with infernal score of 22.6 and with loss of 5’ acceptor stem), and *Helianthus annuus* (*trn*T-GGU gene predicted as pseudo copy with infernal score 21.8), whereas *Barnadesia lehmannii* was selected from the subfamily Barnadesioideae, which showed the presence of the functional copy of the *trn*T-GGU gene with infernal score of 65.8.

## Plant collection and deposition of voucher specimens to herbarium

The publicly available genomics sequences were taken from the National Center for Biotechnology Information. None of the plants was collected and sequenced in the current study. Hence, permission for plant collection and submission to herbarium under a voucher specimen are not applicable.

## Supplementary Information

Supplementary Information 1.

Supplementary Table S1.

## Data Availability

The publicly available data set of genomic sequences was retrieved from the National Center for Biotechnology Information (NCBI) and analyzed in the current study. All accession numbers are provided in the manuscript. The analyses are included in the main manuscript or in the supplementary tables/figures.
