# Supplementary material for: Pseudogenization of the chloroplast threonine (trnT-GGU) gene in the sunflower family (Asteraceae)
Source: Sci Rep. 2021 Oct 26;11:21122. doi: 10.1038/s41598-021-00510-4 (PMC8548347; doi:10.1038/s41598-021-00510-4)

Pseudogenization of the chloroplast threonine (*trn*T-GGU) gene in the sunflower family (Asteraceae)

Abdullah[1, *], Furrukh Mehmood[1], Parviz Heidari[2], Abdur Rahim[3], Ibrar Ahmed[4], Peter Poczai[*5,6,7]

[1]*Department of Biochemistry, Faculty of Biological Sciences, Quaid-i-Azam University, 45320 Islamabad, Pakistan*

[2]*Faculty of Agriculture, Shahrood University of Technology, 3619995161 Shahrood, Iran*

[3]*Government Degree College Nowshera*, Abdul Wali Khan University Mardan, KPK, Pakistan

[4]*Alpha Genomics Private Limited, 45710 Islamabad, Pakistan*

[5]*Finnish Museum of Natural History, P.O. Box 7, FI-00014 University of Helsinki, Finland*

[6]*Faculty of Biological and Environmental Sciences, PO Box 65 FI-00065 University of Helsinki, Finland*

[7]*Institute of Advanced Studies Kőszeg (iASK), PO Box 4, H-9731 Kőszeg, Hungary*

***** Correspondence: *Abdullah (abd.ullah@bs.qau.edu.pk)*

Péter Poczai (peter.poczai@helsinki.fi)

Table S2. Accession numbers of Asteroideae species and prediction of *trn*T-GGU gene

S.No	Species	Accession	tribe	tRNAScan Prediction	Infernal score	Isotype
1	*Ismelia carinata*	MG710387	Anthemideae	Not detected	N/A	N/A
2	*Soliva sessilis*	KX063863	Anthemideae	Pseudo	27.8	Ile2
3	*Ajania pacifica*	MN883841	Anthemideae	tRNAs with mismatch isotypes	27.8	Ile2
4	*Artemisia maritima*	MK532038	Anthemideae	tRNAs with mismatch isotypes	27.8	Ile2
5	*Artemisia ordosica*	NC_046571	Anthemideae	tRNAs with mismatch isotypes	27.8	Ile2
6	*Chrysanthemum boreale*	MG913594	Anthemideae	tRNAs with mismatch isotypes	27.8	Ile2
7	*Chrysanthemum indicum*	JN867592	Anthemideae	tRNAs with mismatch isotypes	27.8	Ile2
8	*Crossostephium chinense*	MH708560	Anthemideae	tRNAs with mismatch isotypes	27.8	Ile2
9	*Leucanthemum virgatum*	MN996243	Anthemideae	tRNAs with mismatch isotypes	27.8	Ile2
10	*Neopallasia pectinata*	MW007388	Anthemideae	tRNAs with mismatch isotypes	27.8	Ile2
11	*Opisthopappus taihangensis*	MK552323	Anthemideae	tRNAs with mismatch isotypes	27.8	Ile2
12	*Tanacetum cinerariifolium*	MT104464	Anthemideae	tRNAs with mismatch isotypes	27.8	Ile2
13	*Aster hersileoides*	MK290823	Astereae	Not detected	N/A	N/A
14	*Aster hypoleucus*	MK290824	Astereae	Not detected	N/A	N/A
15	*Blakiella bartsiifolia*	KX063886	Astereae	Not detected	N/A	N/A
16	*Conyza bonariensis*	KX792499	Astereae	Not detected	N/A	N/A
17	*Erigeron breviscapus*	MK279916	Astereae	Not detected	N/A	N/A
18	*Erigeron canadensis*	MK737940	Astereae	Not detected	N/A	N/A
19	*Eschenbachia blinii*	KX085421	Astereae	Not detected	N/A	N/A
20	*Floscaldasia hypsophila*	KX063916	Astereae	Not detected	N/A	N/A
21	*Heteroplexis incana*	MN172194	Astereae	Not detected	N/A	N/A
22	*Hinterhubera ericoides*	KX063910	Astereae	Not detected	N/A	N/A
23	*Laestadia muscicola*	KX063873	Astereae	Not detected	N/A	N/A
24	*Solidago decurrens*	MT991010	Astereae	Not detected	N/A	N/A
25	*Westoniella kohkemperi*	KX063921	Astereae	Not detected	N/A	N/A
26	*Exostigma notobellidiastrum*	KX063881	Astereae	Not detected	N/A	N/A
27	*Archibaccharis asperifolia*	KX063859	Astereae	Pseudo	15.3	Lys
28	*Aztecaster matudae*	KX063935	Astereae	Thr	22.6	N/A
29	*Baccharis aliena*	KX063869	Astereae	Thr	22.6	N/A
30	*Baccharis genistelloides*	KX063864	Astereae	Thr	22.6	N/A

31	*Diplostephium azureum*	KX063907	Astereae	Thr	22.6	N/A
32	*Diplostephium barclayanum*	KX063865	Astereae	Thr	22.6	N/A
33	*Laennecia sophiifolia*	KX063899	Astereae	Thr	22.6	N/A
34	*Lagenophora cuchumatanica*	KX063879	Astereae	Thr	22.6	N/A
35	*Parastrephia quadrangularis*	KX063923	Astereae	Thr	22.6	N/A
36	*Piofontia alveolata*	KX063856	Astereae	Thr	22.6	N/A
37	*Piofontia antioquensis*	KX063898	Astereae	Thr	22.6	N/A
38	*Symphyotrichum subulatum*	NC_050667	Astereae	Thr	22.6	N/A
39	*Llerasia caucana*	KX063908	Astereae	tRNAs with mismatch isotypes	27.8	Ile2
40	*Nannoglottis ravida*	MT767106	Astereae	tRNAs with mismatch isotypes	27.8	Ile2
41	*Oritrophium peruvianum*	KX063861	Astereae	tRNAs with mismatch isotypes	27.8	Ile2
42	*Chrysanthemoides incana*	This study	Calenduleae	tRNAs with mismatch isotypes	27.8	Ile2
43	*Bidens amplectens*	MN433103	Coreopsideae	Not detected	N/A	N/A
44	*Bidens torta*	NC_047275	Coreopsideae	Not detected	N/A	N/A
45	*Cosmos bipinnatus*	MN518845	Coreopsideae	Not detected	N/A	N/A
46	*Ageratum conyzoides*	MK905238	Eupatorieae	Not detected	N/A	N/A
47	*Dimerostemma asperatum*	MT700540	Eupatorieae	Not detected	N/A	N/A
48	*Iostephane heterophylla*	MT700542	Eupatorieae	Not detected	N/A	N/A
49	*Mikania micrantha*	KX154571	Eupatorieae	Not detected	N/A	N/A
50	*Praxelis clematidea*	KF922320	Eupatorieae	Not detected	N/A	N/A
51	*Ageratina adenophora*	JF826503	Eupatorieae	Pseudo	N/A	N/A
52	*Chromolaena odorata*	NC_050055	Eupatorieae	Pseudo	20.9	N/A
53	*Pappobolus lanatus var. lanatus*	MT700543	Eupatorieae	Pseudo	21.8	N/A
54	*Anaphalis sinica*	KX148081	Gnaphalieae	Not detected	N/A	N/A
55	*Helichrysum italicum*	NC_041458	Gnaphalieae	Not detected	N/A	N/A
56	*Leontopodium leiolepis*	KM267636	Gnaphalieae	Not detected	N/A	N/A
57	*Marshallia caespitosa*	MH037175	Helenieae	Not detected	N/A	N/A
58	*Marshallia grandiflora*	MH037177	Helenieae	Not detected	N/A	N/A
59	*Aldama anchusifolia*	MN337902	Heliantheae	Not detected	N/A	N/A
60	*Ambrosia artemisiifolia*	MF362689	Heliantheae	Not detected	N/A	N/A
61	*Ambrosia trifida*	MG029118	Heliantheae	Not detected	N/A	N/A
62	*Eclipta alba*	MF993496	Heliantheae	Not detected	N/A	N/A

63	*Eclipta prostrata*	KU361242	Heliantheae	Not detected	N/A	N/A
64	*Parthenium hysterophorus*	MT576959	Heliantheae	Not detected	N/A	N/A
65	*Rudbeckia laciniata*	MN518844	Heliantheae	Not detected	N/A	N/A
66	*Sphagneticola calendulacea*	KY828438	Heliantheae	Not detected	N/A	N/A
67	*Sphagneticola trilobata*	KY940274	Heliantheae	Not detected	N/A	N/A
68	*Xanthium sibiricum*	MH473582	Heliantheae	Not detected	N/A	N/A
69	*Echinacea purpurea*	KX548224	Heliantheae	Pseudo	20.9	N/A
70	*Echinacea tennesseensis*	KX548223	Heliantheae	pseudo	20.9	N/A
71	*Helianthus annuus*	DQ383815	Heliantheae	Pseudo	21.8	N/A
72	*Helianthus argophyllus*	KU314500	Heliantheae	Pseudo	21.8	N/A
73	*Tithonia diversifolia*	MT576958	Heliantheae	Pseudo	20.9	N/A
74	*Pluchea indica*	NC_038194	Inuleae	tRNAs with mismatch isotypes; truncated start and end	23	Lys
75	*Achyrachaena mollis*	MF663009	Madieae	Not detected	N/A	N/A
76	*Eriophyllum lanatum*	MH183145	Madieae	Not detected	N/A	N/A
77	*Lasthenia burkei*	KM360047	Madieae	Not detected	N/A	N/A
78	*Lasthenia californica*	KY965816	Madieae	Not detected	N/A	N/A
79	*Galinsoga parviflora*	MK737938	Millerieae	Not detected	N/A	N/A
80	*Galinsoga quadriradiata*	KX752097	Millerieae	Not detected	N/A	N/A
81	*Guizotia abyssinica*	EU549769	Millerieae	Not detected	N/A	N/A
82	*Sigesbeckia orientalis*	MN240004	Millerieae	Not detected	N/A	N/A
83	*Dendrosenecio brassiciformis*	MG560051	Senecioneae	Not detected	N/A	N/A
84	*Dendrosenecio cheranganiensis*	MG560046	Senecioneae	Not detected	N/A	N/A
85	*Farfugium japonicum*	MT929248	Senecioneae	Not detected	N/A	N/A
86	*Gynoxys asterotricha*	MK044798	Senecioneae	Not detected	N/A	N/A
87	*Gynoxys mandonii*	MK056106	Senecioneae	Not detected	N/A	N/A
88	*Jacobaea vulgaris*	HQ234669	Senecioneae	Not detected	N/A	N/A
89	*Ligularia fischeri*	KT988070	Senecioneae	Not detected	N/A	N/A
90	*Ligularia mongolica*	NC_039384	Senecioneae	Not detected	N/A	N/A
91	*Nordenstamia repanda*	MK086040	Senecioneae	Not detected	N/A	N/A
92	*Pericallis hybrida*	KT285537	Senecioneae	Not detected	N/A	N/A
93	*Petasites japonicus*	MN385243	Senecioneae	Not detected	N/A	N/A

94	*Senecio keniophytum*	MH483946	Senecioneae	Not detected	N/A	N/A
95	*Senecio vulgaris*	MK654722	Senecioneae	Not detected	N/A	N/A
96	*Flaveria bidentis*	MK836182	Tageteae	Not detected	N/A	N/A
97	*Tagetes erecta*	MN203535	Tageteae	Not detected	N/A	N/A

Table S3. Accession numbers of species of Carduoideae and prediction of *trn*T-GGU gene

S.No	Species	Accession	tRNAScan Prediction	Infernal score	Isotype	Anticodon
1	*Arctium lappa*	NC_042724	Thr	55.7	Thr	CGU
2	*Atractylodes chinensis*	NC_037484	Thr	57.1	Thr	GGU
3	*Carduus acanthoides*	MK652228	Thr	57	Thr	CGU
4	*Carthamus tinctorius*	MK983238	Thr	57	Thr	CGU
5	*Centaurea diffusa*	KJ690264	Thr	57	Thr	CGU
6	*Cirsium japonicum*	MW035606	Thr	57	Thr	CGU
7	*Cynara cardunculus*	KP842720	Thr	57	Thr	CGU
8	*Dolomiaea calophylla*	MT128668	Thr	57	Thr	CGU
9	*Saussurea inversa*	MT554929	Thr	57	Thr	CGU
10	*Silybum marianum*	KT267161	Thr	57	Thr	CGU
11	*Synurus deltoides*	NC_046830	Thr	57	Thr	CGU

Table S4. Accession numbers of Cichorioideae species and prediction of *trnT-GGU* gene

S.No	Species	Accession	tRNAScan Prediction	Infernal score	Isotype
1	*Cichorium intybus*	MK569377	Thr	34.6	Thr
2	*Crepidiastrum lanceolatum*	MK358413	Thr	34.6	Thr
3	*Dendroseris berteroana*	MK371014	Thr	34.6	Thr
4	*Gymnanthemum amygdalinum*	MT795180	Thr	33.7	Thr
5	*Hypochaeris radicata*	MH746729	tRNAs with mismatch isotypes,truncated start and truncatedend end	20.2	Lys
6	*Lactuca raddeana*	MN402448	Pseudo	29.3	Thr
7	*Lapsanastrum humile*	MK358416	Thr	31	Thr
8	*Reichardia ligulata*	MN893255	Thr	39.6	Thr
9	*Sonchus webbii*	MK033508	Thr	34.6	Thr
10	*Stebbinsia umbrella*	MN822134	Not detected		Thr
11	*Taraxacum amplum*	KX499525	Thr	34.6	Thr
12	*Youngia japonica*	MK358417	Thr	31	Thr
13	Ixeris polycephala	MK358415	Not detected	N/A	N/A

Table S5. Codon usage comparison among the species with functional and non-functional *trn*T-GGU gene

Codon	Amio acid	Number of codons				
		Artemisia ordosica	***Aster hersileoides***	***Symphyotrichum subulatum***	***Helianthus annuus***	***Barnadesia lehmannii***
GCA	A	410	408	407	415	407
GCC	A	229	235	237	233	246
GCG	A	152	160	161	156	158
GCT	A	615	626	619	625	631
TGC	C	87	87	85	79	83
TGT	C	202	208	209	206	215
GAC	D	213	212	210	218	218
GAT	D	857	855	851	836	856
GAA	E	986	981	986	989	998
GAG	E	343	354	354	342	374
TTC	F	499	517	521	510	535
TTT	F	980	982	976	953	979
GGA	G	697	691	694	699	701
GGC	G	190	198	197	188	201
GGG	G	303	324	321	296	306
GGT	G	582	574	576	583	594
CAC	H	146	155	154	148	142
CAT	H	470	457	457	456	481
ATA	I	690	691	679	686	685
ATC	I	434	425	427	453	441
ATT	I	1075	1081	1082	1047	1079
AAA	K	1030	1035	1053	1015	1059
AAG	K	363	368	370	368	362
CTA	L	361	377	380	384	386
CTC	L	194	191	190	188	201
CTG	L	189	182	183	171	178
CTT	L	622	612	607	603	606
TTA	L	882	865	865	854	849
TTG	L	576	580	580	580	598
ATG	M	622	635	629	625	622
AAC	N	283	291	284	281	278

AAT	N	1008	979	975	984	1000
CCA	P	325	319	324	318	312
CCC	P	187	210	205	203	203
CCG	P	160	165	165	166	179
CCT	P	441	410	410	417	408
CAA	Q	733	729	726	699	714
CAG	Q	233	222	222	227	220
AGA	R	482	499	492	484	491
AGG	R	170	174	169	169	187
CGA	R	341	347	354	344	339
CGC	R	104	109	112	100	105
CGG	R	122	115	110	119	136
CGT	R	348	351	347	349	349
AGC	S	122	119	117	113	115
AGT	S	414	403	405	399	407
TCA	S	413	409	406	404	403
TCC	S	323	310	306	302	326
TCG	S	163	167	168	158	191
TCT	S	583	592	592	580	594
ACA	T	412	405	399	400	400
ACC	T	241	240	237	236	247
ACG	T	124	141	142	135	138
ACT	T	527	530	536	519	537
GTA	V	537	525	521	515	534
GTC	V	174	178	177	187	184
GTG	V	187	192	189	187	209
GTT	V	508	510	514	500	506
TGG	W	458	461	457	447	470
TAC	Y	182	176	180	182	182
TAT	Y	800	800	796	789	805
TAA	*	51	50	51	52	47
TAG	*	21	21	21	19	23
TGA	*	15	15	15	16	16

Table S6. The accession numbers of the species of *Artemisia*, *Aldama*, and *Diplostephium*

Serial number	Species	Accession number
1	*Artemisia absinthium*	MK188885
2	*Artemisia annua*	MF623173
3	*Artemisia argyi*	NC_030785
4	*Artemisia argyrophylla*	MF034022
5	*Artemisia capillaris*	NC_031400
6	*Artemisia feddei*	MG951486
7	*Artemisia freyniana f. discolor*	MG951487
8	*Artemisia frigida*	JX293720
9	*Artemisia fukudo*	MK569048
10	*Artemisia gmelinii*	KU736962
11	*Artemisia hallaisanensis*	NC_031399
12	*Artemisia japonica*	KY073390
13	*Artemisia maritima*	MK532038
14	*Artemisia montana*	KF887960
15	*Artemisia nakaii*	NC_02591
16	*Artemisia ordosica*	MN932370
17	*Artemisia princeps*	MF034021
18	*Artemisia rubripes*	MG951496
19	*Artemisia scoparia*	MN385624
20	*Artemisia selengensis*	MH042532
21	*Artemisia sieversiana*	MG951499
22	*Artemisia stolonifera*	MG951500
23	*Aldama anchusifolia*	MN337902
24	*Aldama arenaria*	MN337903
25	*Aldama aspilioides*	MN337905
26	*Aldama bakeriana*	MN337906
27	*Aldama bracteata*	MN337907
28	*Aldama canescens*	MN337908
29	*Aldama corumbensis*	MN337909
30	*Aldama dentata*	MN337910
31	*Aldama filifolia*	MN337890
32	*Aldama fusiformis*	MN337891
33	*Aldama gardneri*	MN337892
34	*Aldama goyazii*	MN337893
35	*Aldama grandiflora*	MN337894
36	*Aldama kunthiana*	MN337895
37	*Aldama linearis*	MN337896
38	*Aldama macrorhiza*	MN337897
39	*Aldama megapotamica*	MN337898
40	*Aldama nudibasilaris*	MN337899
41	*Aldama nudicaulis*	MN337900

42	*Aldama pilosa*	MN337901
43	*Aldama trichophylla*	MN337911
44	*Diplostephium azureum*	KX063907
45	*Diplostephium barclayanum*	KX063865
46	*Diplostephium cajamarquillense*	KX063894
47	*Diplostephium callilepis*	KX063870
48	*Diplostephium cinereum*	KX063889
49	*Diplostephium crypteriophyllum*	KX063905
50	*Diplostephium empetrifolium*	KX063925
51	*Diplostephium ericoides*	KX063892
52	*Diplostephium espinosae*	KX063903
53	*Diplostephium foliosissimum*	KX063909
54	*Diplostephium gnidioides*	KX063887
55	*Diplostephium goodspeedii*	KX063940
56	*Diplostephium haenkei*	KX063893
57	*Diplostephium hartwegii*	KX063880
58	*Diplostephium hippophae*	KX063944
59	*Diplostephium jelskii*	KX063860
60	*Diplostephium juniperinum*	KX063883
61	*Diplostephium lechleri*	KX063868
62	*Diplostephium meyenii*	KX063919
63	*Diplostephium oblanceolatum*	KX063941
64	*Diplostephium oxapampanum*	KX063884
65	*Diplostephium pulchrum*	KX063857
66	*Diplostephium sagasteguii*	KX063932
67	*Diplostephium serratifolium*	KX063924
68	*Diplostephium spinulosum*	KX063917

Figure S1. Structure of *trn*T-GGU gene of species of 13 subfamilies. One species was taken from each subfamily. The *trn*T-GGU gene of *Barnadesia lehmannii* is labeled to show the functional parts as representative of all species. The insertion occurred in the species of four subfamilies of core Asteraceae (Gymnarrhenoideae, Cichorioideae, Corymbioideae, and Asteroideae), which also correspond to mismatches above the anticodon loop. The insertion is highlighted with a box.

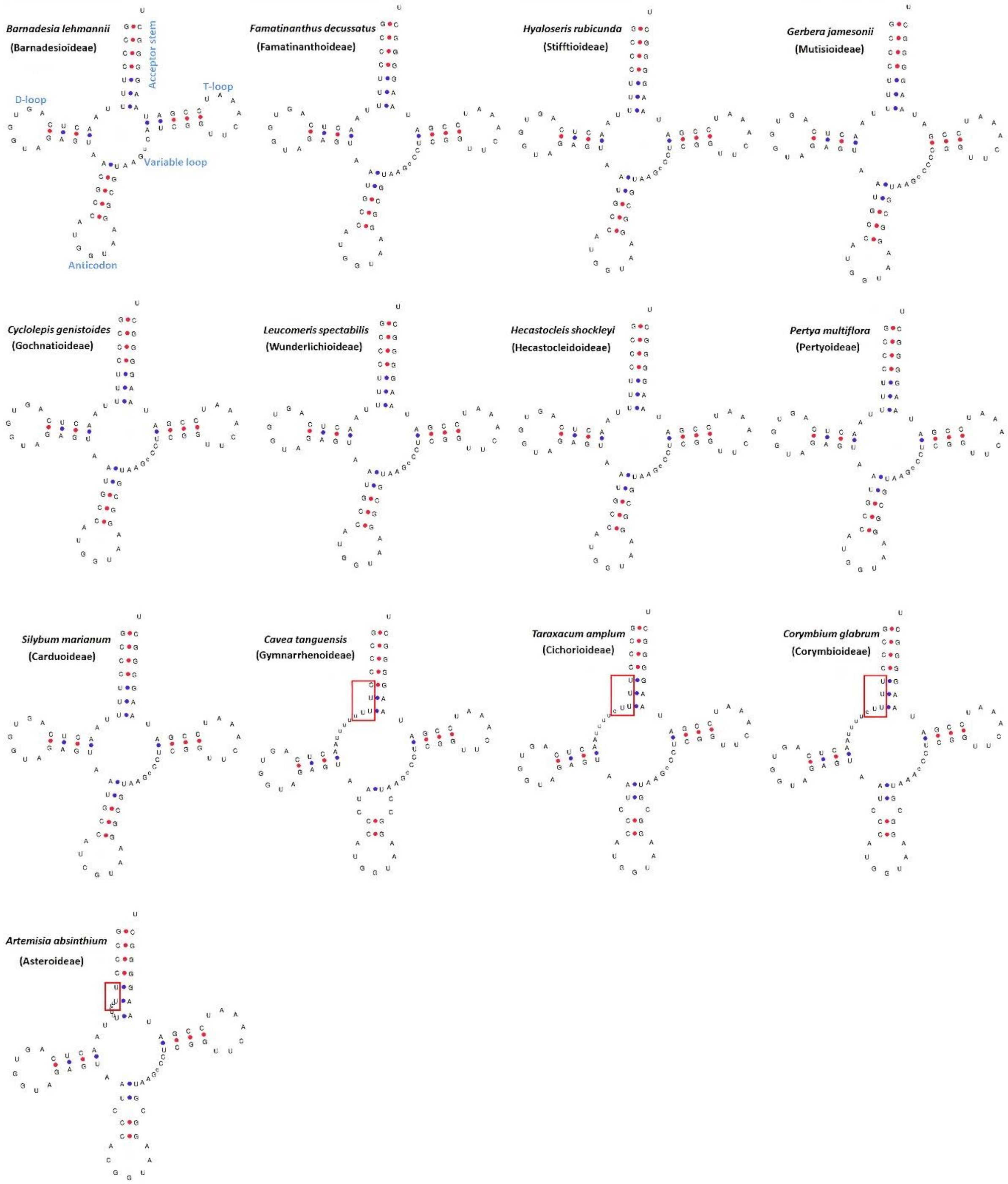

Figure S2. Multiple alignment of *trn*T-GGU gene among the species of the subfamily Carduoideae. The sequence of *Silybum marianum* used as reference.

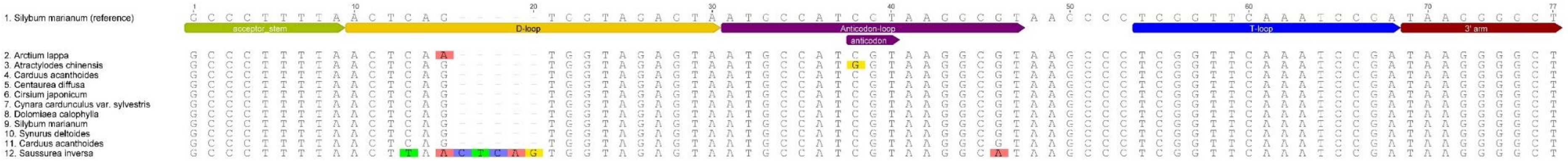

Figure S3. Multiple alignment of *trn*T-GGU gene among the species of the subfamily Cichorioideae. The sequence of *Silybum marianum* used as reference.

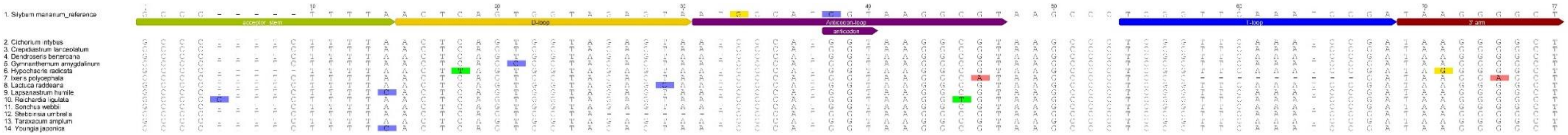

Figure S4. Multiple alignment of *trn*T-GGU gene among the species of the subfamily Asteroideae. The sequence of *Silybum marianum* used as reference.

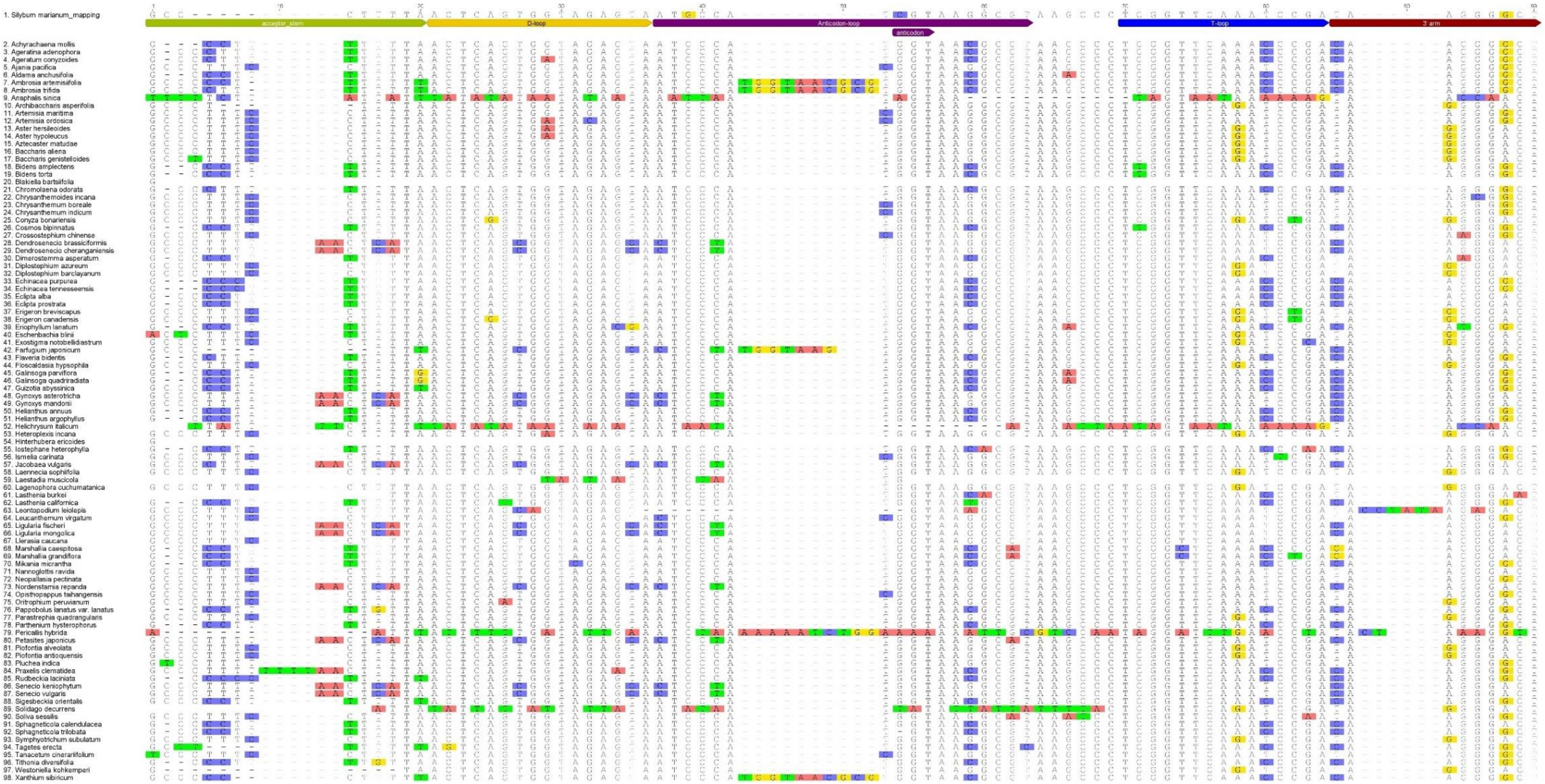

Figure S5. Multiple alignment of *trn*T-GGU gene among the species of the species of *Artemisia*. The sequence of *Silybum marianum* used as reference.

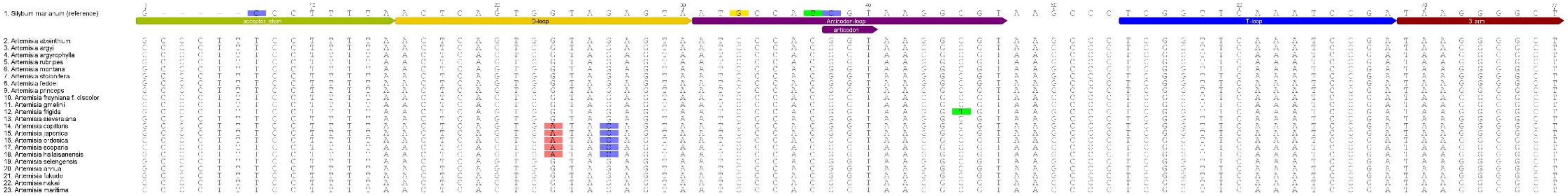

Figure S6. Multiple alignment of *trn*T-GGU gene among the species of the species of *Aldama*. The sequence of *Silybum marianum* used as reference.

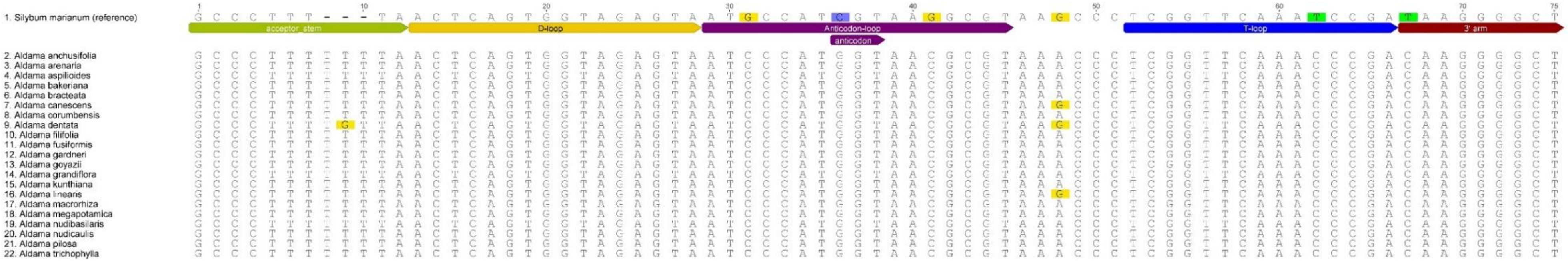

Figure S7. Multiple alignment of *trn*T-GGU gene among the species of the species of *Diplostephium.* The sequence of *Silybum marianum* used as reference.

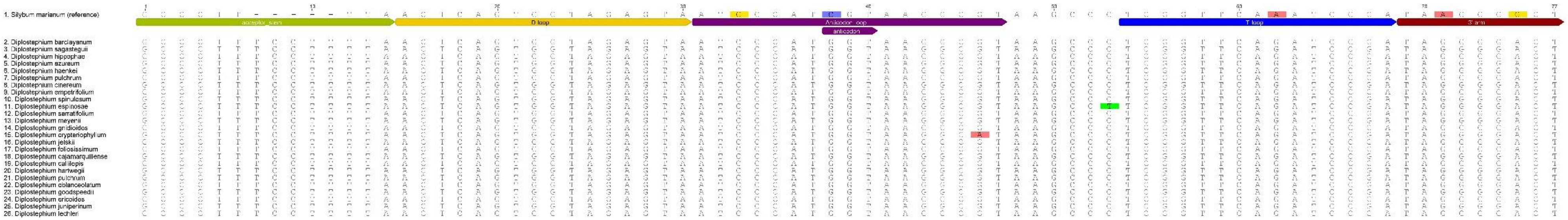

Supplement: Supplementary file 1 — Supplementary Information 1. [file 41598_2021_510_MOESM1_ESM.pdf]
